# Evaluation of human nonmercaptalbumin as a marker for oxidative stress and its association with various parameters in blood

**DOI:** 10.3164/jcbn.17-5

**Published:** 2017-07-20

**Authors:** Rie Masudo, Keiko Yasukawa, Takahiro Nojiri, Naoyuki Yoshikawa, Hironori Shimosaka, Shinji Sone, Yumiko Oike, Akemi Ugawa, Tsutomu Yamazaki, Kentaro Shimokado, Yutaka Yatomi, Hitoshi Ikeda

**Affiliations:** 1 Department of Clinical Laboratory Medicine, Graduate School of Medicine, The University of Tokyo, 7-3-1 Hongo, Bunkyo-ku, Tokyo 113-8655, Japan; 2 Department of Geriatrics and Vascular Medicine, Tokyo Medical and Dental University Graduate School, 1-5-45 Yushima, Bunkyo-ku, Tokyo 113-8519, Japan; 3 Department of Center for Epidemiology & Preventive Medicine, Graduate School of Medicine, The University of Tokyo, 7-3-1 Hongo, Bunkyo-ku, Tokyo 113-8655, Japan

**Keywords:** human nonmercaptalbumin, oxidative stress, HPLC

## Abstract

Oxidative status of albumin was not a useful biomarker for oxidative stress in practical use due to time-consuming measuring method. We evaluated oxidized, human nonmercaptalbumin measured more quickly than ever by a novel method using anion-exchange HPLC. In 60 subjects taking a general health examination, mean serum human nonmercaptalbumin level was 25.1 ± 3.0% with no gender difference but positive correlation with age. There were no links between human nonmercaptalbumin and C-reactive protein, γ-glutamyltransferase or iron, reportedly associated with oxidative stress. Human nonmercaptalbumin correlated with systolic blood pressure, pulse pressure and body mass index among physical findings. Positive correlations were observed between human nonmercaptalbumin and AST, LDH, BUN, or creatinine, suggesting that oxidative stress may link with liver injury and renal function. Human nonmercaptalbumin correlated with uric acid in female but not in male, suggesting that higher uric acid levels may be associated with increased oxidative stress only in female. As another gender difference, white blood cell counts correlated with human nonmercaptalbumin in female, while the parameters for red blood cells correlated with human nonmercaptalbumin in male. In conclusion, serum human nonmercaptalbumin level in healthy subjects was approximately 25% as previously reported. Oxidative stress may be closely associated with hypertension, obesity, liver injury, renal function, and anemia.

## Introduction

It is well known that oxidative stress caused by reactive oxygen and free radicals is involved in pathogenesis of various kinds of diseases such as diabetes mellitus, hypertension, hyperlipidemia, arteriosclerosis, periodontal diseases, Alzheimer’s disease, Parkinson’s disease, and cancers.^(1)^ However, no highly sensitive biomarkers have been developed to evaluate the condition of oxidative stress and the treatment effect of antioxidants in the clinical setting.

To this end, we have been interested in albumin, one of the major antioxidants in human serum; albumin is capable of scavenging hydroxyl radicals with its reduced (-SH) cysteine residue (Cys34). As a result, the thiol group of Cys34 in albumin exists in either a reduced (-SH) or oxidized (-S-S-) form; the former is called reduced albumin or human mercaptalbumin (HMA) and the latter, oxidized albumin or human nonmercaptalbumin (HNA). Because albumin is distributed widely in intra- and extra-vascular spaces, the oxidation of albumin reportedly reflects the status of oxidative stress in the whole body. In healthy adults, approximately 70–80% of serum albumin has been shown to be HMA, and the rest, HNA.^(2)^ Although these findings suggest that HMA and HNA could be a reliable blood marker for oxidative stress, the measuring method of HMA and HNA has been problematic, being extremely time-consuming.^(3,4)^ Thus, we have developed a novel method using anion-exchange HPLC system, rendering those measurements more rapid and reliable.

Using this novel method, we evaluated HNA in the subjects who had undergone a general health examination. Then, because it has been reported that some clinical parameters in the blood, such as C-reactive protein (CRP),^(5)^ γ-glutamyltransferase (GGT)^(6)^ or uric acid (UA),^(7)^ might be indicative of oxidative stress, we examined whether these parameters would be really associated with oxidative stress and could predict its status.

## Methods

### Subjects and data collection

Participants were recruited at the time of a health examination performed at the University of Tokyo Hospital between June and July in 2016. Venous blood sampling after an overnight fast was performed as part of a health check-up. Chemistry and complete blood count test were performed. This study was carried out in accordance with the ethical guidelines of the 1975 Declaration of Helsinki and was approved by the Institutional Research Ethics Committee of the Faculty of Medicine at the University of Tokyo. Written informed consent was obtained for the use of samples.

### Measurement of HMA and HNA

We used the LabSolutions system (Shimadzu Co., Ltd., Kyoto, Japan), consisting of a degasser (DGU20A3R), two pumps (LC-20AT), an auto-sampler (SIL30AC), a thermostatic oven (CTO-20AC), a fluorescence detector (RF-20Axs) and a system controller (CBM-20A).

The gel and the column for the analysis of HMA and HNA were made using the following method. A polyvinyl alcohol cross-linked gel (9 µm in diameter) (Asahipak GS-520; Asahi Kasei Co., Ltd., Tokyo, Japan) was dried in a vacuum for more than 16 h and then suspended in 10 ml of dimethyl sulfoxide (Tokyo Chemical Industry Co., Ltd., Tokyo, Japan) per 1 g of dried gel. Next, 20 mmol of epichlorohydrin for each 1 g of dried gel was added to suspend the gel; the reaction was allowed to proceed for 20 h at 30°C.

The activated gel was filtrated and reacted with a 10% aqueous solution of diethyl amine (Wako Pure Chem. Ind., Ltd., Osaka, Japan) for 20 h. The synthesized anion-exchange gel was packed in a stainless column (50 × 7.6 mm I.D.).

Using the above system and column, the optimal HPLC conditions, which consisted of the compositions and concentrations of eluents A and B, pH, flow rate, gradient condition of the eluents (ethanol concentration, magnesium concentration, and pH), temperature of the thermostatic oven, and sample with the goal of establishing a rapid and convenient method of measuring HMA and HNA. All the reagents were either HPLC grade or a special grade (Wako Pure Chem.). The conditions for fluorescence detection were the same as those previously reported by Hayashi *et al.*, and the excitation and emission wavelengths were 280 nm and 340 nm, respectively.^(8,9)^

The conventional method using anion-exchange chromatography developed by Era *et al.*^(4)^ uses a three-step gradient program of 1%, 3%, and 10% ethanol concentrations for eluents, and the analysis conditions are complicated; the method takes about 1 h to perform, including time for column equilibration. And it is not applicable in clinical settings requiring the measurement of large numbers of samples simultaneously. Thus, we established a simple and rapid HPLC method suitable for clinical measurements with an analytical time of only 12 min per test. The repeatability (within-day variability) and reproducibility (day-to-day variability) were 0.30% and 0.27% (CV), respectively.

### Statistical analysis

Data were expressed as mean ± SD. Mann-Whitney *U* test was used to make a comparison between male and female. Regression equations and correlation coefficients between HNA and the categorical variables were determined by Spearman’s rank correlation coefficient. A *p* value <0.05 was considered statistically significant.

### Ethical approval

This study was carried out in accordance with the ethical guidelines of the 1975 Declaration of Helsinki and was approved by the Institutional Research Ethics Committee of the Faculty of Medicine at the University of Tokyo (Approval number 2565-(2)). Written informed consent was obtained for the use of samples.

## Results and Discussion

### Characteristics of the subjects and their serum HNA levels

**Fig. 1:**
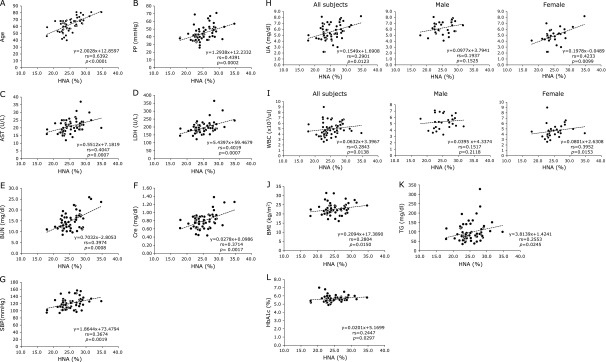
Relationships between HNA and clinical parameters which showed significant positive correlation with HNA. (A) Relationship between HNA and age (*n* = 60). (B) Relationship between HNA and PP (*n* = 60). (C) Relationship between HNA and AST (*n* = 60). (D) Relationship between HNA and LDH (*n* = 60). (E) Relationship between HNA and BUN (*n* = 60). (F) Relationship between HNA and Cre (*n* = 60). (G) Relationship between HNA and SBP (*n* = 60). (H) Relationship between HNA and UA in all subjects (*n* = 60), male (*n* = 30) and female (*n* = 30). (I) Relationship between HNA and WBC in all subjects (*n* = 60), male (*n* = 30) and female (n = 30). (J) Relationship between HNA and BMI (*n* = 60). (K) Relationship between HNA and TG (*n* = 60). (L) Relationship between HNA and HbA1C (*n* = 60).

Characteristics of the subjects are shown in Table 1. The mean age was 63.1 ± 9.9, suggesting that the cohort analyzed consisted of relatively older subjects. Judged by the measured mean values of various parameters, the enrolled subjects were assumed to be essentially healthy. In those subjects, the mean serum HNA level was 25.1 ± 3.0%. Serum HNA levels in male were 25.1 ± 3.1%, and not different from those in female (25.0 ± 3.2%). On the other hand, there was a positive correlation between HNA and age, as previously reported (Fig. 1A).^(10)^ The previous report indicated that approximately 20–30% of serum albumin was HNA,^(2)^ and our current result obtained with the applicants for a health examination is in line with this previous finding.

### Relationships between serum HNA levels and various clinical parameters

Then, the relationships between serum HNA levels and various clinical parameters were evaluated, and the results are shown in Table 2. The significant positive correlation with HNA was observed in pulse pressure (PP), AST, LDH, BUN, Cre, systolic blood pressure (SBP), UA, WBC, BMI, TG, and HbA1c (Table 2 and Fig. 1B–L). The gender differences regarding the correlation with HNA are noted in AST, Cre, and UA.

Although CRP, GGT and Fe have been previously shown to be associated with oxidative stress, there were no links observed between those factors and HNA. Because GGT has a gender difference, its link with HNA was analyzed in male and in female, respectively, but the close link was not found. On the other hand, a significant correlation was observed between GGT and Fe (rs = 0.3802, *p* = 0.0014) but not between CRP and GGT, and CRP and Fe. Thus, CRP, GGT and Fe should be re-evaluated as a marker for oxidative stress, importantly with measurement of total antioxidant capacity.^(11)^

Roles of oxidative stress in hypertension have been demonstrated.^(12)^ In line with this report, PP and SBP among blood pressure-related parameters correlated strongly with HNA, while DBP did not. Of note is the report that the treatment with anti-oxidant, vitamin C, lowered SBP and PP but not DBP in the elderly.^(13)^ The current result may be in line with this previous report. Nonetheless, it should be clarified why oxidative stress correlated only with SBP.

Obesity reportedly increases oxidative stress especially in female,^(14)^ and notably BMI correlated well with HNA similarly in male and in female in the current study (Table 2). Regarding blood pressure and obesity, it should be elucidated whether their associations with oxidative stress would be cause or effect.

As a daily habit, HNA was not different between drinker and non-drinker (Table 3). Although smoking history did not affect HNA, either, whether present smoking might increase HNA was not known because of the small number of subject with smoking (*n* = 5; Table 3).

Highly positive correlations were observed between HNA and BUN or Cre (Table 2, Fig. 1E and F), suggesting a close correlation between oxidative stress and renal functions. The rapid alteration of redox state of human serum albumin was previously reported before and after hemodialysis.^(15)^ Furthermore, HMA was reportedly decreased in patients with chronic kidney diseases.^(16)^ Thus, renal functions may be one of the major determinants of serum HNA levels and hence the status of oxidative stress.

UA is formed from xanthine as one of purine bases by xanthine oxidase (XO), when nucleic acid is metabolized. Thus, hyperuricemia may be often associated with high activity of XO, and the abundant XO could cause the production of free radicals. As a result, hyperuricemia could lead to active generation of oxidative stress.^(17)^ In contrast, because UA can work as a scavenger against reactive oxygen, UA may play a role as an antioxidant, suggesting that hyperuricemia could cause reduction of reactive oxygen.^(18)^ Thus, there have been antithetic concepts whether higher UA levels could be associated with increased or decreased oxidative stress. In the current study, although the significant positive correlation was observed between HNA and UA, the strong positive correlation was determined only in female but not in male (Table 2 and Fig. 1H), suggesting that higher UA levels may be associated with increased oxidative stress in female but not in male. Of interest is the fact that higher UA levels reportedly had a lower future risk of developing Parkinson’s disease in male but not in female,^(19)^ being consistent with the result of current study.

It has been known that neutrophils, once activated, could release reactive oxygen species, contributing to endothelial damage in cardiovascular disease.^(20)^ On the other hand, monocytes are crucial cells in the generation of atherosclerotic lesions, because they, when stimulated, can adhere to endothelium, causing cardiovascular injury.^(21)^ Moreover, it has been reported that the oxidative stresses of neutrophils are affected by HbA1c.^(22)^ In line with these previous findings, WBC correlated with HNA especially in female. On the other hand, the parameters for red blood cells did correlate with HNA in male but not in female. The link between anemia and oxidative stress may be explained by the aging of hematopoietic stem cells caused by reactive oxygen species, which may result in hematopoietic disorder.^(23)^ In male, platelet counts correlated with HNA but not in female, suggesting that hematopoiesis in general may be more susceptible to reactive oxygen species in male than in female.

As to the parameters for liver injury, AST but not ALT and LDH had a link with HNA closer in female. Thus, liver as well as kidney may be one of the important target organs by oxidative stress. In line with this, albumin was shown to be severely oxidized in acute-on-chronic liver failure,^(24)^ and HNA was reportedly a prognostic marker in chronic liver failure.^(25)^

In conclusion, oxidized, HNA level in serum, measured by a novel method, was approximately 25% in healthy subjects, correlated with age but not with gender, in line with previous reports. Based on the results from regression analyses with clinical parameters, hypertension, obesity, liver injury, renal function, and anemia may be closely associated with oxidative stress.

## Figures and Tables

**Table 1:** Characteristics of the subjects

	All (*n* = 60)	Male (*n* = 30)	Female (*n* = 30)	*p* value
Age	63.1 ± 9.9	64.7 ± 9.4	61.4 ± 10.2	0.083
Human nonmercaptalbumin (HNA) (%)	25.1 ± 3.0	25.1 ± 3.1	25.0 ± 3.2	0.379
Body Mass Index (BMI) (kg/m^2^)	22.6 ± 3.0	23.5 ± 2.1	21.7 ± 3.6	0.001
Systolic blood pressure (SBP) (mmHg)	120.2 ± 15.4	120.4 ± 16.3	120.0 ± 14.8	0.471
Diastolic blood pressure (DBP) (mmHg)	75.6 ± 11.0	74.6 ± 11.8	76.5 ± 10.3	0.273
Pulse pressure (PP) (mmHg)	44.7 ± 9.6	45.8 ± 10.0	43.5 ± 9.2	0.195
γ-glutamyltransferase (GGT) (U/L)	28.9 ±21.7	35.6 ± 28.0	22.1 ± 8.6	0.001
Alkaline phosphatase (ALP) (U/L)	179.7 ± 50.5	171.0 ± 30.3	188.4 ± 64.1	0.095
Lactate dehydrogenase (LDH) (U/L)	195.8 ± 37.8	188.5 ± 29.7	203.1 ± 43.8	0.127
Cholineesterase (CHE) (U/L)	316.1 ± 55.1	321.5 ± 40.3	310.8 ± 66.9	0.175
Aspartate aminotransferase (AST) (U/L)	21.0 ± 4.4	21.8 ± 3.3	20.2 ± 5.2	0.031
Alanine aminotransferase (ALT) (U/L)	17.6 ± 6.5	19.2 ± 6.0	16.0 ± 6.7	0.008
Amylase (AMY) (U/L)	72.8 ± 27.0	73.3 ± 29.6	72.2 ± 24.7	0.436
Total protein (TP) (g/L)	6.9 ± 0.3	6.9 ± 0.3	6.9 ± 0.3	0.480
Albumin (ALB) (g/L)	4.2 ± 0.2	4.2 ± 0.2	4.1 ± 0.2	0.023
Total bilirubin (TB) (mg/dl)	0.9 ± 0.2	0.9 ± 0.3	0.9 ± 0.2	0.250
Creatinine (Cre) (mg/dl)	0.80 ± 0.19	0.90 ± 0.14	0.69 ± 0.17	<0.001
Blood urea nitrogen (BUN) (mg/dl)	14.8 ± 4.0	15.1 ± 3.6	14.5 ± 4.3	0.160
Uric acid (UA) (mg/dl)	5.6 ± 1.3	6.3 ± 1.1	4.9 ± 1.3	<0.001
Total cholesterol (TC) (mg/dl)	204.7 ± 28.1	200.6 ± 23.0	208.8 ± 32.2	0.140
Triglyceride (TG) (mg/dl)	97.0 ± 51.2	108.2 ± 58.7	85.9 ± 40.4	0.043
Iron (Fe) (µg/dl)	103.4 ± 31.3	109.3 ± 30.1	97.5 ± 31.9	0.147
C-reactive protein (CRP) (mg/dl)	0.31 ± 1.82	0.08 ± 0.11	0.53 ± 2.58	0.114
Carcinoembryonic antigen (CEA) (pg/ml)	2.6 ± 1.3	2.7 ± 1.5	2.5 ± 1.2	0.236
Glucose (GLU) (mg/dl)	96.1 ± 12.7	98.9 ± 14.2	93.3 ± 10.6	0.077
Hemoglobin A1c (HbA1c) (%)	5.7 ± 0.4	5.8 ± 0.5	5.6 ± 0.3	0.102
Red blood cell (RBC) (×10^12^/L)	4.52 ± 0.44	4.67 ± 0.46	4.37 ± 0.38	0.002
White blood cell (WBC) (×10^9^/L)	5.0 ± 1.2	5.3 ± 1.1	4.6 ± 1.3	0.007
Platelet (PLT) (×10^9^/L)	224.7 ± 51.1	210.8 ± 37.1	238.6 ± 59.4	0.049
Hemoglobin (HGB) (g/dl)	13.9 ± 1.4	14.6 ± 1.1	13.2 ± 1.2	<0.001
Hematocrit (HCT) (%)	41.8 ± 3.7	43.4 ± 3.0	40.1 ± 3.7	0.001

Date are presented as mean ± SD, and compared by Mann-Whitney *U* test.

**Table 2:** Spearman’s rank correlation coefficient (rs) between HNA and the categorical variables

	All (*n* = 60)		Male (*n* = 30)		Female (*n* = 30)	
	rs	*p* value	rs	*p* value	rs	*p* value
Age	0.6392	<0.0001	0.6281	0.0001	0.6154	0.0001
PP	0.4391	0.0002	0.4003	0.0142	0.5028	0.0023
AST	0.4047	0.0007	0.1726	0.1809	0.6077	0.0002
LDH	0.4019	0.0007	0.3512	0.0285	0.4609	0.0052
BUN	0.3974	0.0008	0.3699	0.0221	0.3962	0.0151
Cre	0.3714	0.0017	0.5550	0.0007	0.2875	0.0617
SBP	0.3674	0.0019	0.3476	0.0299	0.4207	0.0103
UA	0.2901	0.0123	0.1937	0.1525	0.4233	0.0099
WBC	0.2843	0.0138	0.1517	0.2118	0.3952	0.0153
BMI	0.2804	0.0150	0.2467	0.0944	0.2850	0.0634
TG	0.2553	0.0245	0.2810	0.0663	0.2650	0.0785
HbA1c	0.2447	0.0297	0.2221	0.1191	0.1873	0.1609
TP	0.2105	0.0532	0.1124	0.2771	0.3366	0.0345
PLT	–0.1932	0.0695	–0.3255	0.0396	–0.0923	0.3137
ALT	0.1731	0.0929	–0.0046	0.4904	0.2358	0.1048
HGB	–0.1419	0.1397	–0.4675	0.0046	–0.0232	0.4515
HCT	–0.1389	0.1450	–0.3849	0.0178	–0.0716	0.3534
DBP	0.1232	0.1742	0.1720	0.1818	0.1459	0.2208
GGT	0.1203	0.1799	–0.0944	0.3099	0.1634	0.1941
CRP	0.1128	0.1955	0.1448	0.2227	0.0515	0.3934
Fe	–0.0884	0.2508	0.1467	0.2197	–0.0513	0.3939
RBC	–0.0841	0.2615	–0.3064	0.0498	–0.0961	0.3067
CEA	–0.0622	0.3184	–0.0441	0.4085	0.1127	0.2767
AMY	0.0597	0.3252	0.2240	0.1171	0.1359	0.2370
CHE	0.0509	0.3497	–0.1234	0.2580	0.2344	0.1062
TB	–0.0481	0.3575	–0.0961	0.3067	–0.0656	0.3653
ALP	0.0460	0.3634	0.1220	0.2604	0.0245	0.4489
GLU	–0.0083	0.4749	–0.1994	0.1454	0.1951	0.1507
ALB	0.0056	0.4831	0.0024	0.4950	–0.0073	0.4848
TC	0.0020	0.4940	0.2058	0.1377	–0.1443	0.2234

**Table 3:** HNA according to BMI, blood pressure, and daily habits

	*n*	HNA (%)	*p* value
BMI			
<25	50	24.8 ± 3.0	0.098
≥25	10	26.5 ± 3.6	
SBP			
<130	46	24.6 ± 3.0	0.013
≥130	14	26.7 ± 3.1	
DBP			
<85	45	25.0 ± 3.2	0.321
≥85	15	25.4 ± 2.9	
PP			
≤50	46	24.3 ± 2.7	0.002
>50	14	27.5 ± 3.2	
Smoking history			
Nonsmoker	39	25.2 ± 3.1	0.293
Ever smoker/smoker	16/5	24.9 ± 3.3	
Drinking history			
Nondrinker	29	25.1 ± 3.2	0.497
Drinker	31	25.1 ± 3.1	

Date are presented as mean ± SD, and compared by Mann-Whitney *U* test.

## Abbreviations

ALB: albumin
AMY: amylase
BUN: blood urea nitrogen
CHE: cholineesterase
CRE: creatinine
DBP: diastolic blood pressure
GGT: γ-glutamyltransferase
GLU: glucose
HMA: human mercaptalbumin
HNA: human nonmercaptalbumin
LDH: lactate dehydrogenase
PP: pulse pressure
SBP: systolic blood pressure
TB: total bilirubin
TC: total cholesterol
TG: triglyceride
TP: total protein
